# A case of SAPHO syndrome: a DISH of radiological features to be considered

**DOI:** 10.1186/s43166-022-00159-5

**Published:** 2022-12-15

**Authors:** Fakhreddin Sabooniha

**Affiliations:** Sabzevar University of Medical Sciences, Sabzevar, 96137-95143 Iran

**Keywords:** SAPHO syndrome, Palmoplantar pustulosis, Psoriasis, DISH

## Abstract

**Background:**

The acronym SAPHO stands for synovitis, acne, pustulosis, hyperostosis, and osteitis. It is a rare heterogenous disease with unknown etiology and a chronic relapsing and remitting course^1^. Its skin and osteoarticular manifestations including palmoplantar pustulosis (PPP) and synovitis may be transient which further complicate the diagnosis. So, awareness about all features of the syndrome throughout the time seems mandatory for correct diagnosis and avoidance of unnecessary procedures.

Case presentation.

A case of SAPHO syndrome being reported in a middle-aged man who presented with bilateral PPP and exacerbation of back pain which developed shortly after covid-19 vaccine injection with a history of more than 20 years of inflammatory thoracic back pain and psoriasis vulgaris who initially had been worked up for metastatic bony lesions based on radiologic studies, irrespective of his skin lesions. The patient had good response to alendronate 70 mg weekly and celecoxib 200 mg BID without aggravation of existing skin lesions or new psoriatic lesions.

**Conclusion:**

This case report aims to inform rheumatologists and radiologists about various radiologic and dermatologic manifestations of SAPHO syndrome with emphasizing on taking into consideration of past and present skin lesions in the interpretation of the radiologic signs in order to prevent irrelevant procedures or hazardous imaging and to urge rheumatology societies to set up a SAPHO registry for future randomized controlled trials. Suggestion of PPP responsiveness to NSAIDs as a new potential diagnostic tool for SAPHO diagnosis is another goal. It also aims to point out the possible coexistence of SAPHO and SpA or DISH syndrome.

## Background

SAPHO syndrome is a rare heterogenous disease with an unknown etiology and estimated prevalence of less than 1/10,000 [1]. It affects mainly individuals in the 4th to 6th decades of life. The acronym SAPHO represents synovitis, acne, pustulosis, hyperostosis, and osteitis, a variety of conditions which are not necessarily simultaneously being occurred. It is a clinicoradiologic term which was coined by Chamot et al. in 1987 to better explain and classify the patients presented with aforementioned signs [2]. The disease has a chronic and relapsing course with overall good prognosis [3]. Occasionally, there is a prolonged time interval between musculoskeletal and dermatologic manifestations which could precede each other, a condition that further complicates its correct and timely diagnosis. The most commonly affected osteoarticular site in SAPHO syndrome is the anterior chest, followed by the spine, most frequently thoracic vertebrae. In the majority of cases, spine lesions are segmental, involving two to four adjacent vertebrae [4]. Palmoplantar pustulosis (PPP) is the most prevalent cutaneous manifestation followed by acne, pustular psoriasis, and rarely hidradenitis suppurativa which all are characterized with neutrophilic infiltration [5]. The disease has some shared features with seronegative spondyloarthritides such as inflammatory back pain, synovitis, enthesitis, non-marginal and rarely marginal syndesmophytes, and bone erosions. Also its relationship with inflammatory bowel disease has been suggested, but more prevalent HLA-B27 haplotype in this population was not confirmed [6]. Nevertheless, development of massive paravertebral bridging osteophytes makes it somewhat similar to other proliferative bone disorders such as diffuse idiopathic skeletal hyperostosis (DISH) [6]. A case of ossification of the posterior longitudinal ligament of the cervical spine has also been reported in association with SAPHO syndrome [7]. The question if “this syndrome a bridging entity between spondyloarthritides and proliferative bone diseases or not” remains to be elucidated. While the precise pathogenesis of SAPHO syndrome is unclear, it is understood that immune dysfunction, infections, and genetic susceptibilities may contribute to its development [8]. The diagnostic yield of imaging modalities varies based on the ostedestructive or osteoproliferative stages of the disease. Treatment options are empiric and based on retrospective reports and case series [5]. Surprisingly, despite using NSAIDs as a first-line therapy for most musculoskeletal presentations of SAPHO syndrome and with regarding NSAIDs as the traditional precipitating factors of psoriasis, there are not notable reports about exacerbation of existing skin lesions or inducing new psoriatic ones in SAPHO patients (unlike other forms of psoriasis) which probably is in part due to different nature of the palmoplantar pustular variant compared to chronic plaque psoriasis. Some of SAPHO patients have only PPP without clinical evidence of osteoarticular involvement despite of concurrent imaging abnormalities [4]. Thus, prompt response of psoriatic skin lesions to NSAIDs may be utilized as a clinical diagnostic tool for this subset of patients, and incorporating it into current diagnostic criteria of SAPHO syndrome would optimize their accuracy.

## Case report

### A case of SAPHO syndrome: a DISH of radiological features to be considered

**Fig. 1:**
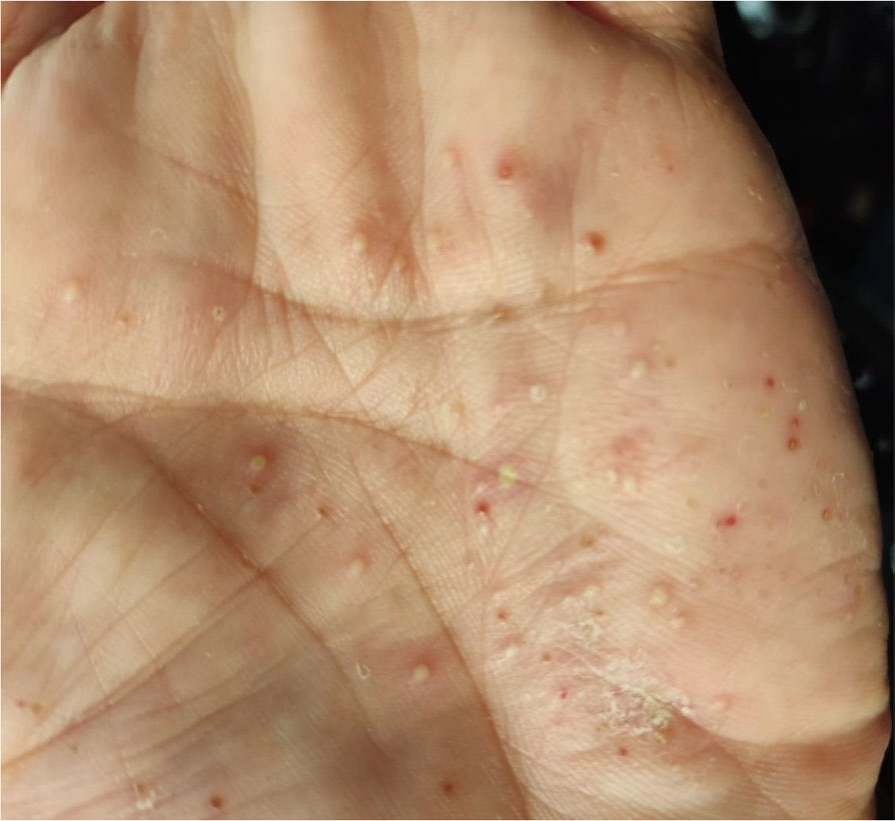
Palmoplantar pustulosis (PPP). Small monomorphic pustules on both palms which erupted shortly after receiving first dose of covid-19 vaccine. Image 1 was obtained via Xiaomi Poco X3 Pro cellphone camera

**Fig. 2:**
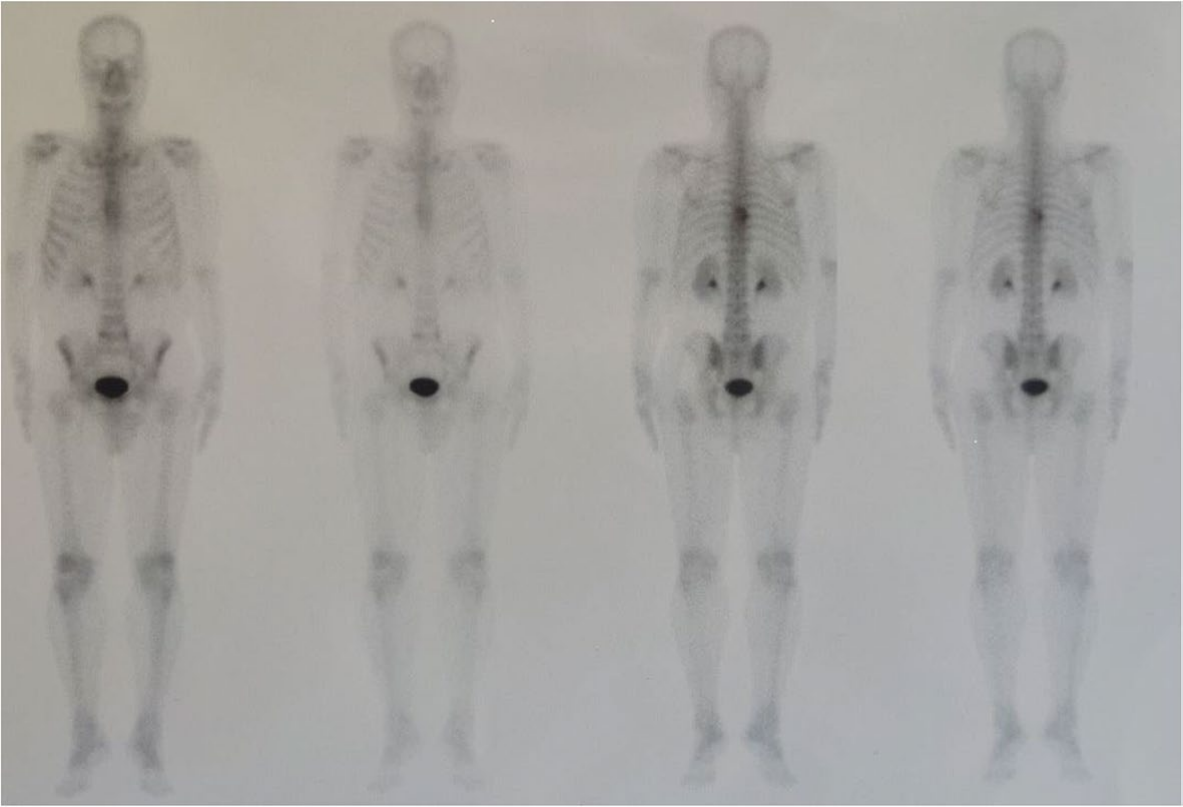
Whole-body bone scintigraphy using Tc-99 MDP showed increased tracer uptake in T7 and T8 vertebrae. Tc-99 MDP, technetium 99-methyl diphosphonate. Image 2 was obtained via Samsung Galaxy Note ultra 5G cellphone camera

**Fig. 3:**
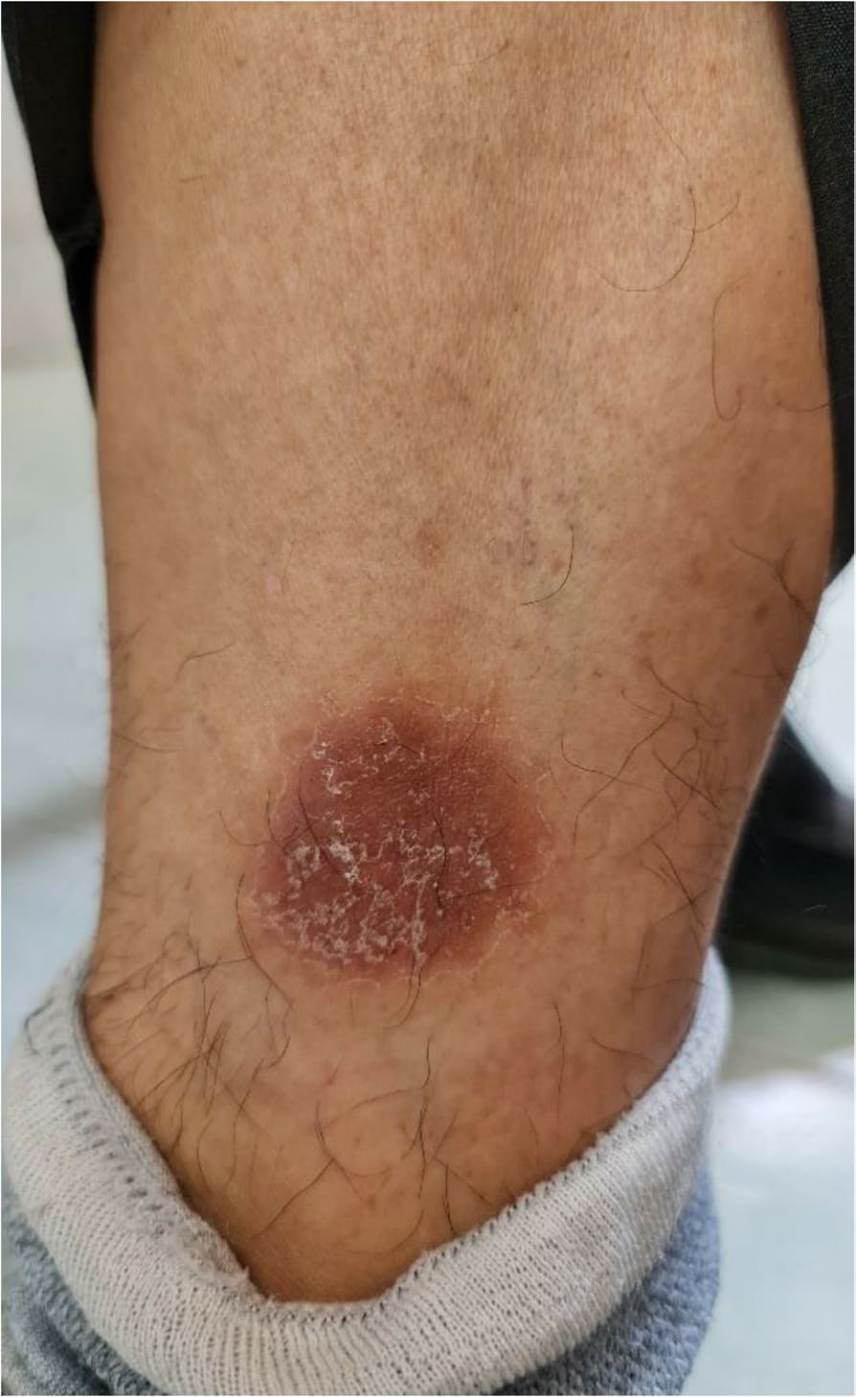
Psoriasis vulgaris. Well-defined red-brown scaly plaque on the lateral aspect of right shin consistent with psoriasis vulgaris. Image 3 was obtained via Samsung Galaxy Note ultra 5G cellphone camera

**Fig. 4:**
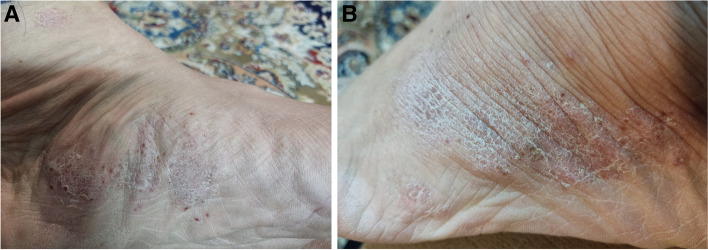
Palmoplantar pustular psoriasis. Scaly plaque with punctuated appearance on the plantar (**A**) and dorsolateral surface of left foot suggestive of palmoplantar pustular psoriasis (**B**). Images 4 A and B were obtained via Xiaomi Poco X3 Pro cellphone camera

**Fig. 5:**
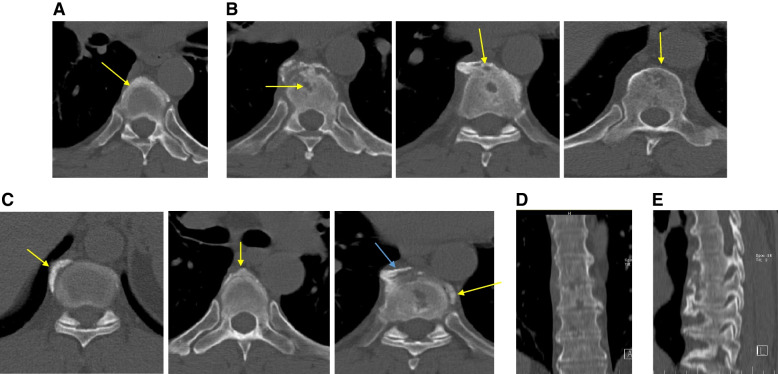
Sagittal and coronal CT of thoracic spines. Coronal view shows markedly cortical thickening (hyperostosis) of thoracic vertebrae (**A**) and multiple cortical erosions (**B**). Severely thickened ossification of anterolateral spinal ligament along with large osteophytes *without disk space involvement* suggestive of DISH (**C**). AP view showing relative symmetrical non-marginal syndesmophytes (**D**). Sagittal view shows thick flowing osteophytes consistent with DISH (**E**). CT, computed tomography; AP, anteroposterior; DISH, diffuse idiopathic skeletal hyperostosis. All radiographic images were screenshotted from the compact discs.

**Fig. 6:**
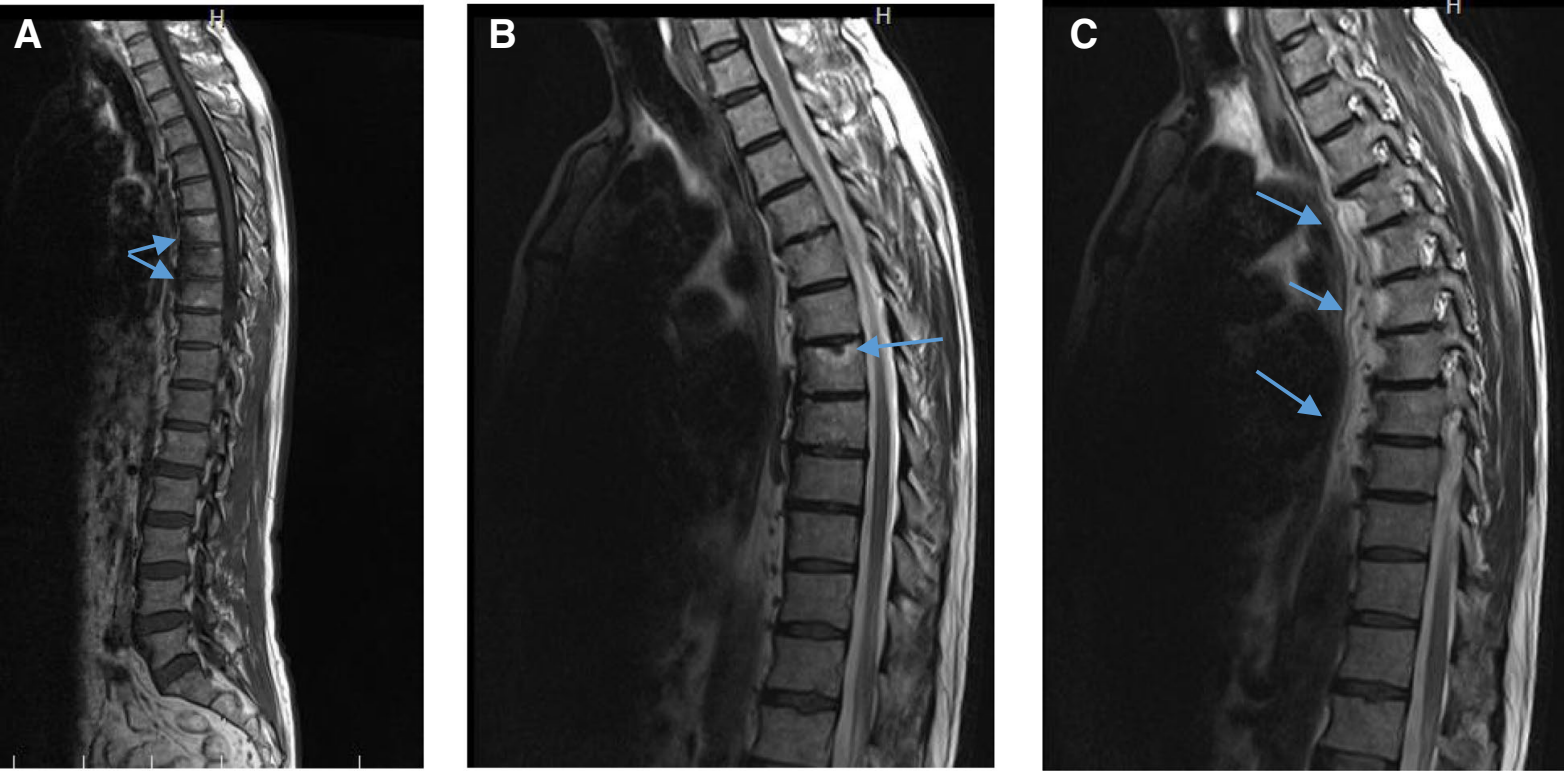
T1- and T2-weighted sagittal spinal MRI. T2-weighted sagittal MRI reveals bone marrow edema (osteitis) of 7th and 8th thoracic vertebrae in parallel with bone scan findings (**A**). T1-weighted MRI showing cortical erosion with surrounding edema in 7th thoracic spinal vertebra (**B**). Flowing thick osteophytes along anterior border of thoracic vertebrae without intervertebral disk involvement consistent with DISH (**C**). MRI, magnetic resonance imaging; DISH, diffuse idiopathic skeletal hyperostosis. All radiographic images were screenshotted from the compact discs.

A 59-year-old man presented to a private orthopedic clinic with exacerbation of his chronic thoracic back pain along with emergence of small monomorphic pustules distributed on both palms and soles consistent with palmoplantar pustulosis (PPP), 9 days after receiving the first dose of covid-19 vaccine (Sinopharm) (Fig. 1). The patient had been suffering from a relapsing–remitting low back pain with variable intensity from 20 years ago which was inflammatory in nature without seeking medical attention. Two weeks later, a magnetic resonance imaging (MRI) is requested by orthopedic specialist which showed bone marrow edema and erosions in the 7th and 8th thoracic vertebrae. Markedly elevated erythrocyte sedimentation rate (ESR) and mildly elevated C-reactive protein (CRP) had been reported. Based on MRI findings, a probable metastatic bony process was suspected, and tumor markers including carcinoembryonic antigen, alpha-fetoprotein, CA-125, and CA 19–9 were requested by his treating physician that were reported normal. Irrespective of his skin findings, a diagnostic bone biopsy had been recommended that the patient refused to do the biopsy. Three weeks later, a bone scintigraphy scan was performed which revealed increased radiotracer uptake in T7 and T8 vertebrae without any other abnormality (Fig. 2). Also, a computed tomography (CT) of thoracolumbar spines was performed which demonstrated osteolytic lesions, and subsequently, a bone biopsy is suggested again. Diclofenac 50 mg BID had been started with dramatic pain relief and resolution of skin lesions in contrast to usual cases of psoriasis vulgaris that may be exacerbated with NSAIDs. Because of significant response to diclofenac, the patient did not perform biopsy and presented to our department for further medical consult. The patient also stated mild chronic pain in the right elbow consistent with enthesitis as well as mild chronic pain in the right sacroiliac joint in association with morning stiffness lasting about 15 min. On examination, there was a well-defined red-brown scaly plaque measuring about 3 × 3 cm on the lateral aspect of his right shin suggestive of psoriasis vulgaris (Fig. 3). Also, scaly dry plaques with punctuated appearance on left palmar and dorsolateral aspect of left foot consistent with chronic palmoplantar pustular psoriasis were seen (Fig. 4 A and B). There were no evidence of pitting on fingernails or dactylitis on his hands. The right elbow was mildly tender without any soft tissue swelling or redness. The sternoclavicular and remainder joints were unremarkable. He was not smoker and did not use alcohol or illicit drugs. A repeat laboratory study showed a significant decrease in ESR and a normal CRP. Serum level of uric acid was normal. HLA-B27 was negative. CT showed marked cortical thickening (hyperostosis) and sclerosis, multiple bone erosions, intense ossification of the anterolateral spinal ligament with large osteophytes consistent with diffuse idiopathic skeletal hyperostosis (DISH), and relatively symmetric non-marginal syndesmophytes in thoracic vertebrae (Fig. 5 A–E). MRI of thoracic vertebrae revealed bone marrow edema (osteitis) and erosions of thoracic vertebrae as well as DISH (Fig. 6 A–C). By consideration of MRI, CT, and bone scintigraphy findings and based on his past medical history of psoriasis vulgaris and a recently developed palmoplantar pustulosis, a diagnosis of SAPHO syndrome was made based on diagnostic criteria proposed by Kahn, modified in 2003. The patient had also a diabetes mellitus history, and his mother had been diagnosed with an unspecified rheumatologic disease. Alendronate 70 mg weekly and celecoxib 200 mg BID started, and after 3-month follow-up, palmoplantar pustules had not been recurred, and the plaque on the left shin became dusky and inactive without any new lesions. Coexistence of DISH with SAPHO syndrome is one of the most interesting aspects of this case. This association may be coincidental probably due to more prevalence of DISH in diabetes mellitus type 2 or might be the result of the shared proliferative nature of both diseases. Another important finding was prompt response of both skin and osteoarticular presentations to NSAIDs which may have clinical implications as discussed earlier. Other noticeable presentation of this patient was the eruption of PPP shortly after covid-19 vaccination which further supports the role of antigenic stimulation in its pathogenesis. There are reports of exacerbation of background autoimmune disorders such as psoriatic arthritis and rheumatoid arthritis as well as transient new autoimmune diseases such as adjuvant-induced autoimmune syndrome in the literature. In a large real-world study supported by the European League Against Rheumatism (EULAR) covid-19 database (83% mRNA vaccines), vaccine-related adverse events were observed in 37% of patients, and flare was reported in 4.4% of patients with rheumatic diseases. Another study reported cases of musculoskeletal inflammatory manifestations including synovitis, tenosynovitis, enthesitis, and inflammatory spinal pain with serological evidence of inflammation appearing within 4 weeks of the administration of the first or second dose of one of the covid-19 vaccines. Interestingly, two patients also had inflammatory back pain with active sacroiliitis and spondylitis on MRI. Autoantibodies comprised a rare finding, and HLA-B27 was positive in 31.8% of patients [9]. Meanwhile, this case indicates the importance of interpreting bone lesions in the context of past and present skin findings for correct diagnosis and avoiding unnecessary and invasive procedures such as biopsy and ionizing radiation.

SAPHO syndrome is a rare heterogenous disease with an unknown etiology and estimated prevalence of less than 1/10,000 [1]. It affects mainly individuals in the 4th to 6th decades of life. The disease has a chronic and relapsing course with overall good prognosis [3]. The most common skin manifestation is palmoplantar pustulosis followed by psoriasis vulgaris and acne. Dermatologic manifestations may precede, follow, or occur simultaneously with osteoarticular manifestations, and occasionally, there is a long period of time between dermatologic and rheumatic presentations which further complicate its diagnosis, as in this case [4]. Its rheumatic manifestations include spondylitis, nonerosive synovitis, and enthesitis. It has some common features with spondyloarthritides and proliferative bone disease such as DISH. A challenging issue is discriminating axial psoriatic arthritis (PsA) from SAPHO spondylitis. Antonio Leone et al. (2014) in their review article concluded that paravertebral ossification in SAPHO syndrome is mainly non-marginal and asymmetrical syndesmophytes are similar to but not identical with the syndesmophytes seen in psoriasis. Referring to Sugimoto et al. study, they stated that the radiographic appearance of such ossifications may at first simulate syndesmophytes, but their progression was found to be indicative of new periosteal ossification. They also indicated that other investigators never observed true syndesmophytes and considered these ossifications as enthesophytes rather than true syndesmophytes [10]. Beside, Kahn in his modified criteria for SAPHO diagnosis in 2003 requires coexistence of PPP and psoriasis vulgaris in addition to bone or joint involvement for SAPHO diagnosis which is in apparent contrast to primary proposed criteria in 1994 which considered any sterile osteitis associated with PPP or psoriasis vulgaris as a sufficient criteria for the diagnosis of SAPHO syndrome that could misdiagnose any axial PsA as SAPHO syndrome [11]. Coexistence of PPP and psoriasis vulgaris and the absence of the nail pitting and dactylitis, as well as HLA-B27 negativity, all argue against the PsA as a culprit cause of axial involvement in this case. But what about SAPHO and DISH? DISH course begins between the third and fifth decade of life with a slightly male predominance. It may be associated with synovitis, enthesitis, and more importantly non-marginal syndesmophytes which are also common in SAPHO which make distinguishing between two disorders more difficult [12]. However, the absence of disk space involvement in DISH is an important discriminating feature which in this case favors the coexistence of both SAPHO and DISH. Due to initiation of low back pain in the 4th decade of his life and well-established radiological findings in current studies, it seems reasonable to attribute some of the patient’s symptoms to DISH not to the SAPHO syndrome only. Etiopathogenesis of SAPHO is considered multifactorial. Low virulent pathogens, such as *Propionibacterium acnes*, may trigger an exaggerated inflammatory response of the bone marrow in genetically susceptible individuals, leading to a form of “reactive osteitis.” Its diagnosis is based on clinical and radiologic findings, and yet, there is no consensus on its standard treatment.

## Conclusion

SAPHO is a clinicoradiologic term which all of its presentations do not necessarily occur simultaneously. It is now widely accepted as a distinct entity without a consistent and unified diagnostic criteria and a standardized therapeutic algorithm. Its rarity is partly due to underdiagnosis, and so, there is an unmet need to inform rheumatologist, dermatologists, and other pertaining specialties about its various and complex presentations and to modify its diagnostic criteria by introducing new clinical tools such as responsiveness of skin lesions to NSAIDs in order to fill out the gap between clinical and radiologic manifestations by conducting randomized prospective clinical trials. Also, searching for distinguishing features of SAPHO, DISH, and PsA has important therapeutic and prognostic implications.

## Data Availability

Not applicable.

## Abbreviations

SAPHO: Synovitis, acne, pustulosis, hyperostosis, and osteitis
PPP: Palmoplantar pustulosis
DISH: Diffuse idiopathic skeletal hyperostosis
CT: Computed tomography
MRI: Magnetic resonance imaging
ESR: Erythrocyte sedimentation rate
CRP: C-reactive protein
HLA-B27: Human leukocyte antigen B-27
NSAIDs: Nonsteroid anti-inflammatory drugs
PsA: Psoriatic arthritis
